# Nanozymes-recent development and biomedical applications

**DOI:** 10.1186/s12951-022-01295-y

**Published:** 2022-02-22

**Authors:** Xiangyi Ren, Dongxu Chen, Yan Wang, Huifang Li, Yabing Zhang, Hongying Chen, Xi Li, Minfeng Huo

**Affiliations:** 1 Core Facilities of West China Hospital, Sichuan University, Chengdu, 610041 China; 2 Present Address: Department of Anesthesiology, West China Hospital of Sichuan University, Chengdu, 610041 China; 3 Shanghai Tenth People’s Hospital, Shanghai Frontiers Science Center of Nanocatalytic Medicine, School of Medicine, Tongji University, Shanghai, 200072 People’s Republic of China; 4 State Key Laboratory of High Performance Ceramics and Superfine Microstructure; Research Unit of Nanocatalytic Medicine in Specific Therapy for Serious Disease, Chinese Academy of Medical Sciences (2021RU012), Shanghai Institute of Ceramics Chinese Academy of Sciences, Shanghai, 200050 People’s Republic of China

**Keywords:** Nanozyme, Oxidative stress, Reactive oxygen species, Disease therapy

## Abstract

Nanozyme is a series of nanomaterials with enzyme-mimetic activities that can proceed with the catalytic reactions of natural enzymes. In the field of biomedicine, nanozymes are capturing tremendous attention due to their high stability and low cost. Enzyme-mimetic activities of nanozymes can be regulated by multiple factors, such as the chemical state of metal ion, pH, hydrogen peroxide (H_2_O_2_), and glutathione (GSH) level, presenting great promise for biomedical applications. Over the past decade, multi-functional nanozymes have been developed for various biomedical applications. To promote the understandings of nanozymes and the development of novel and multifunctional nanozymes, we herein provide a comprehensive review of the nanozymes and their applications in the biomedical field. Nanozymes with versatile enzyme-like properties are briefly overviewed, and their mechanism and application are discussed to provide understandings for future research. Finally, underlying challenges and prospects of nanozymes in the biomedical frontier are discussed in this review.

**Graphical Abstract:**

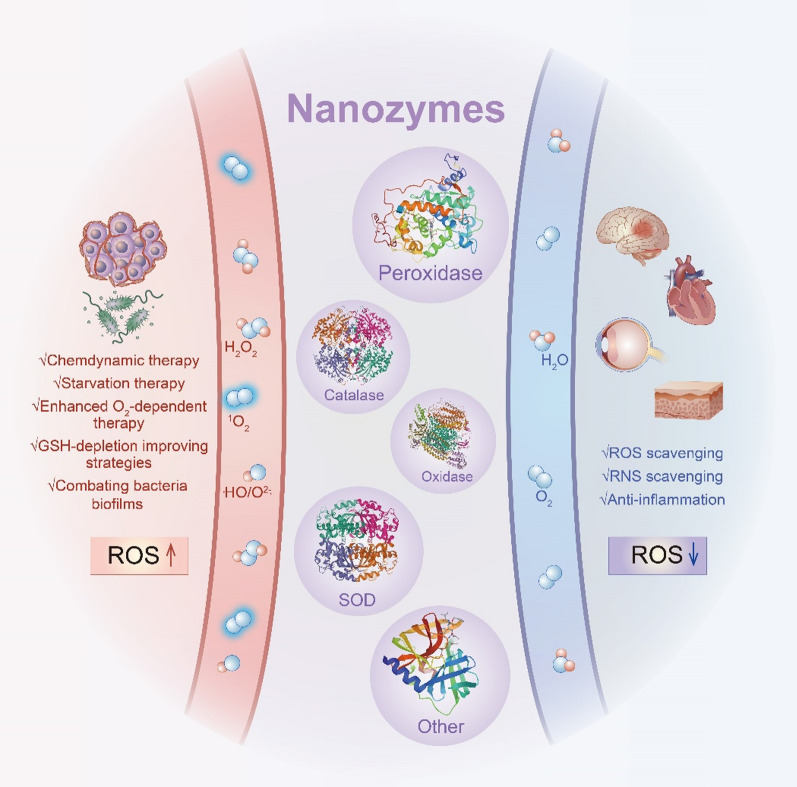

## Background

Nanozymes, as artificial enzymes, are nanomaterials with enzyme mimetic activities, which have attracted considerable interest due to their relatively higher physiochemical stability against harsh environments, higher durability, and lower costs than natural enzymes [1]. In the past decades, numerous nanomaterials have been revealed to elucidate the oxidase (OXD), glucose oxidase (GOD), peroxidase (POD), catalase (CAT), superoxide dismutase (SOD), and glutathione peroxidase (GPx) mimicking activities with extensive biomedical applications [2, 3]. At present, nanozymes are mainly composed of metal and metal oxides, since the metallic active center can effectively mimic the catalytic electronic redox process enabled by natural enzymes. Specifically, the enzyme-mimicking activities are affected by various factors, such as the oxidation states of the metallic center, reduction agent, temperature, and pH in the surrounding environment [4, 5]. Interestingly, the disease features differ from normal tissues provide typical therapeutic options for rational design and application of nanozymes in biomedicine. It is well known that the tumor microenvironment (TME) exhibits higher redox potential levels than the normal tissues. Such characteristics in the tumor can catalyze enzyme-like activities of the nanozymes [6–8]. For instance, metallic ions (such as Fe^3+^, Cu^2+^, and Mn^4+^, etc.) can be reduced to lower-valent metallic ions (Fe^2+^, Cu^+^, and Mn^3+^) by intracellular GSH [9–11]. Hence, the POD activities and catalytic efficiency of the nanozymes could be altered remarkably in the specific pathological microenvironment.

Although numerous nanozymes having been made in the biomedicine field, it is still challenging to obtain a fundamental insight into the key factors that affect the catalytic performance, enzymatic-likes properties, as well as the substrate selectivity of nanozymes, on the basis of the interplay between intrinsic structure and extrinsic environment [12, 13]. Moreover, the catalytic mechanisms of metal oxide nanozymes are pivotal to rationally design novel nanozymes with inherent catalytic capacities and this approach has been widely applied in biomedicine as a controllable multifunctional platform [14, 15]. Recently, many types of nanoparticles with inherent catalytic properties have been reported to achieve various biomedical applications, including oxygen-dependent tumor therapy, radiotherapy, chemodynamic therapy, bacterial infection diseases, and reactive oxygen species (ROS)-related diseases, etc. [16–20]. Therefore, recent advances in the field of nanozyme’s biomedical application may bring new insights into the popularization of nanoparticles in the treatment of the biomedical field.

**Scheme 1:**
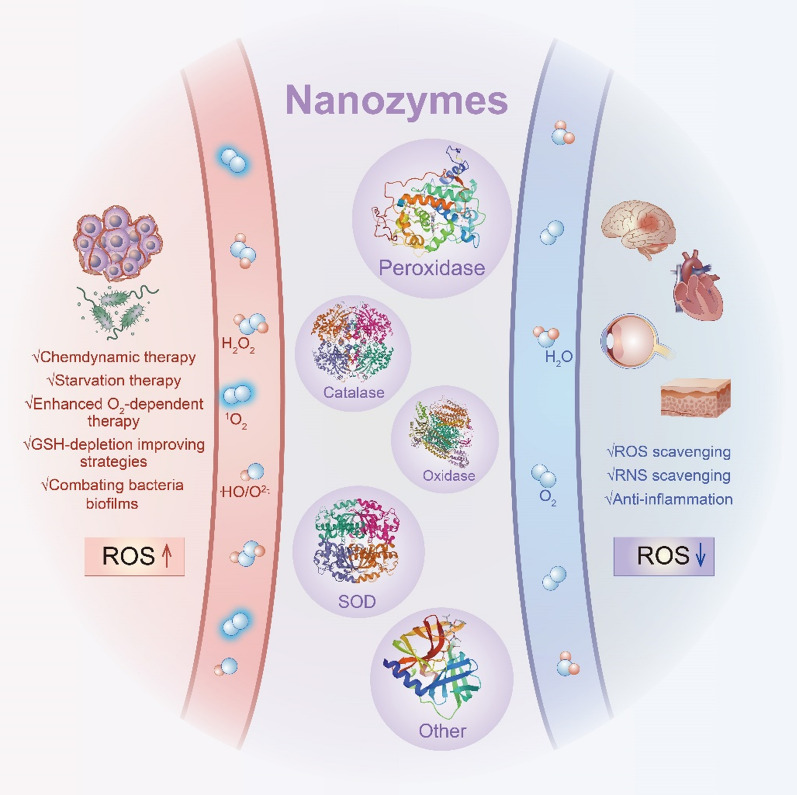
Schematic illustration of metal-based nanozymes for biomedical application

In this review, Different metal- or metal-based nanozymes have been overviewed and described as classified according to their catalytic active center, which significantly impacts the functionalities and activities of the nanozymes during certain catalytic reactions. The versatile enzymes-like properties, mechanism of nanozymes, and the factors that affect the catalytic performance are initially summarized. Then, recently administrated strategies of nanozymes in the therapeutic frontier have been introduced (Scheme 1). Finally, the current challenges of the development of nanozymes and prospects are discussed. We hope that the present review will be of significant benefit for different biomedical fields and provide insightful ideas for the design and development of nanozymes.

## Cerium-based nanozymes

Cerium (Ce)-based nanoparticles have been exploited for biomedical applications since they exhibit multiple enzyme-like activities such as catalase- (CAT), peroxidase- (POD), cytochrome c oxidase-, and superoxide dismutase-mimetic (SOD) functions [21–23]. The underlying mechanism of nanoceria-mediated enzymatic reactions was associated with the chemical state of the cerium element. The reduction state (Ce^3+^) and oxidation state (Ce^4+^) affect the enzyme-like performance of CeO_2_ [24, 25]. Singh et al. reported that CeVO_4_ nanoparticles exhibited cytochrome c oxidase (CcO) activity, which can dismutase oxygen into water at physiological pH conditions due to the electron transfer between Ce^3+^, Ce^4+^, and V^5+^ [26]. Interestingly, the ratio of Ce^3+^/Ce^4+^ of CeVO_4_ has been shown to affect the CcO-like activity of CeVO_4_. The authors suggested that the lower ratio Ce^3+^/Ce^4+^ had higher CcO-like activity in CeVO_4_ while the higher ratio of lowered valence states (Ce^3+^) corresponds to the higher SOD-like activity. The present study demonstrated the enzyme-mimetic activities of Ce-based nanozymes associated with available oxidation states. Importantly, recent studies manifested that the surface defect characteristics of CeO_2_ could partly affect the enzyme-like capability. Recently, Wang et al. revealed the SOD- and CAT mimicking mechanisms of CeO_2_ by first-principles calculations [27]. Their results suggested that oxygen vacancies played critical roles to scavenge superoxide anion (O_2_^•−^) and hydrogen peroxide (H_2_O_2_). The oxygen vacancies impacting enzyme-mimic activities were mainly ascribed to the reduced activation energy and the formation of the intermediate species. This research suggests that the reduction of activation energy by CeO_2_ is critically important in exploring the catalytic processes. Their catalytic activity is substantially affected by the intrinsic properties (e*.*g*.*, dimensions, oxygen vacancy) and physiological factors such as pH, GSH, and temperature. Meanwhile, the oxygen vacancy concentrations were highly dependent on the particle size of CeO_2_ [28].

**Fig. 1:**
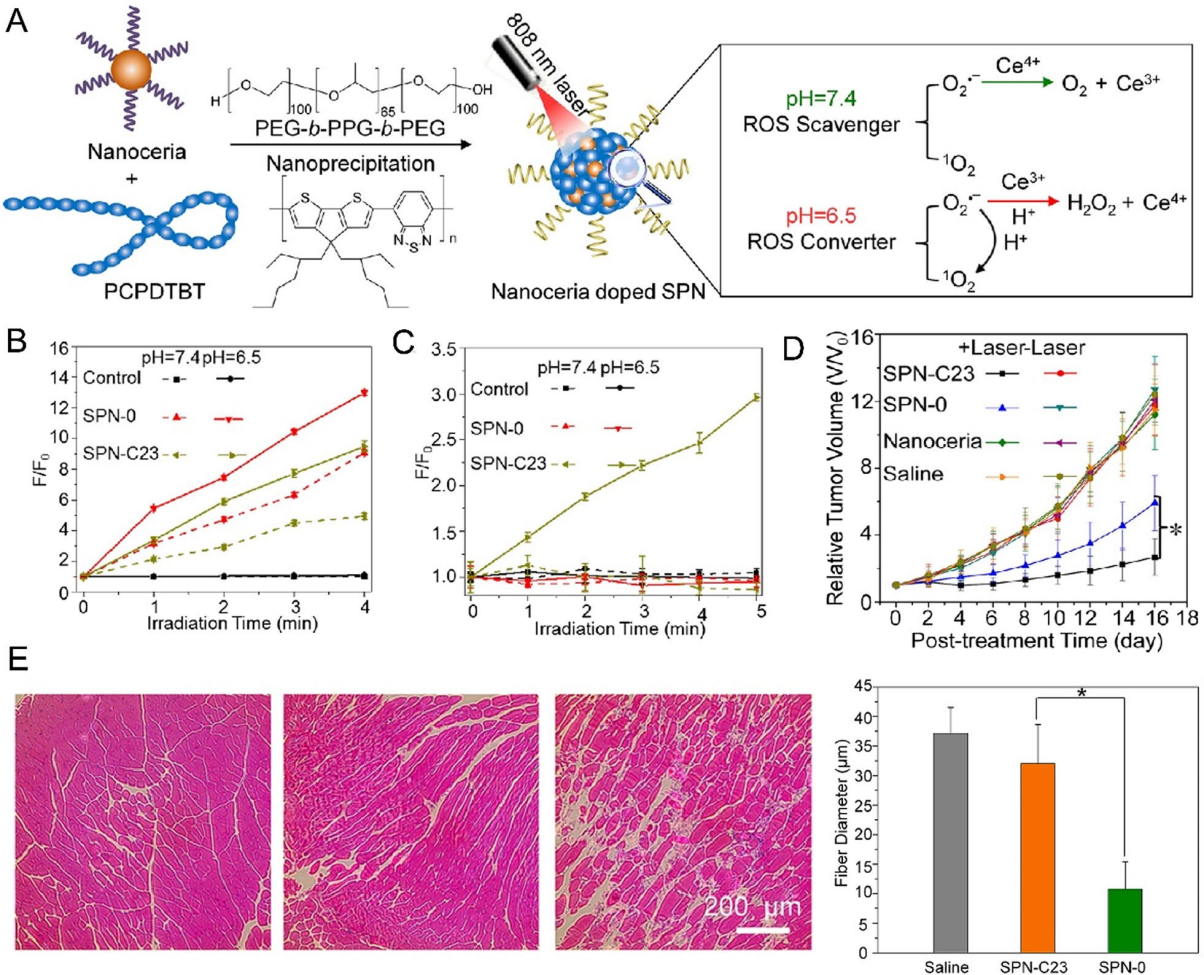
**A** Scheme for nanoceria of self-regulated photodynamic properties at various tissue physiological conditions. **B** SOSG detects ^1^O_2_ production of control, SPN-0, and SPN-C23 under 808 nm laser irradiation. **C** HRP/Amplex Red detects H_2_O_2_ generated at pH = 6.5 or pH = 7.4 under NIR laser irradiation. **D** Relative tumor growth curves after intravenous injection of saline and varied treatment groups. **E** H&E staining for evaluated tissues damaged after PDT [31]. Copyright 2017 American Chemical Society

Despite the advancements made in biomedicine, nanoceria has key limitations that need to be overcome. For instance, the precise regulation of the enzyme activities remains challenging for nanoceria to meet the biomedical application. Moreover, the toxicity of nanoceria against normal tissues still presents great challenges in achieving clinical application. Previous studies have explored the toxicity effect of CeO_2_ of varied shapes in RAW264.7 cell line. Compared with the cubic/octahedral morphological nanoparticles, the rod-like CeO_2_ shows increasing serum concentration of tumor necrosis factor alpha (TNF-α) and lactate dehydrogenase (LDH) release, demonstrating the morphological-dependent cytotoxicities [29]. The biocompatibility of CeO_2_ nanoparticle was demonstrated in further in vivo studies in rats when administrating CeO_2_ of as high as 20 mg/kg [30]. These observations provide an important inspiration for other nanozyme to achieve higher therapeutic effect with minimal toxicity. To solve the low selectively and poor therapeutic effects, it is highly appealing to design controllable nanoceria-based therapeutic systems. Noteworthily, the catalytic behavior of nanoceria is also determined by physiologically catalytic environments. By controlling enzyme activities in a TME or light stimuli, a desired therapeutic effect with low tissue damage could be achieved. Zhu et al*.* reported that the self-regulated nanoceria-doped poly-(cyclopentadithiophene-alt-benzothiadiazole) (SPN-C23) as smart nanoplatforms for tumor photodynamic therapy (PDT) [31]. When near-infrared (NIR) laser was irradiated against the tumor tissue with acidic microenvironment (pH = 6.5), SPN-C23, acted as a ROS converter, was able to amplify PDT damage against tumor tissues through catalyzing O_2_^•−^ to produce H_2_O_2_ (Fig. 1A). When exposed to the normal microenvironment (pH = 7.4), Ce^4+^ of SPN-C23 could transform O_2_^•−^ to O_2_ with generated Ce^3+^ due to oxygen vacancies in the surface of nanoceria. Consequently, SPN-C23 exhibited high singlet oxygen sensor green (SOSG) fluorescence enhancement at pH = 6.5 and possessed relatively low fluorescence intensity at pH = 7.4 (Fig. 1B), demonstrating that pH-dependent single oxygen species (^1^O_2_) production of SPN-C23. SPN-C23 also possesses higher fluorescence at pH = 6.5 with NIR laser irradiation, indicating that the generation of H_2_O_2_ (Fig. 1C). PDT efficiency of SPN-C23 was evaluated on in vivo 4-T1 xenograft tumor model. Under NIR laser irradiation, the growth of the tumor in SPN-C23 treated group was significantly suppressed after intravenous injection of SPN-C23 for 16 days (Fig. 1D). Tissue damage by SPN-0 and SPN-C23 were examined by histological (H&E) staining respectively. The damaged area of the healthy muscle tissues in the SPN-C23 group was significantly decreased as compared to the SPN-0 treatment group (Fig. 1E). Their study provides an effective approach by utilizing ceria-based nanozyme to regulate PDT against cancer with high biocompatibility.

**Fig. 2:**
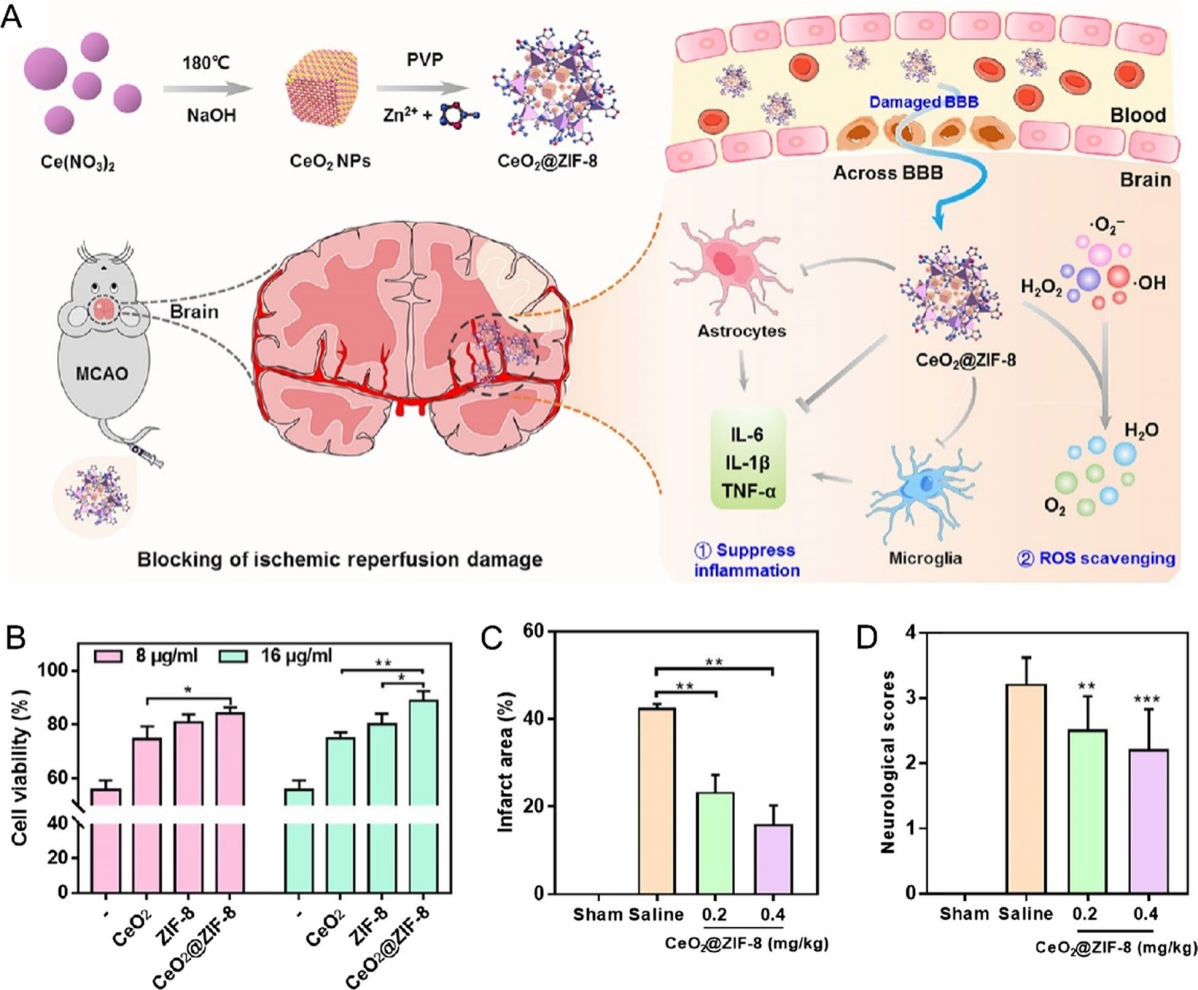
**A** Fabrication methodologies and therapeutic mechanism of CeO_2_@ZIF-8 nanozymes. **B** The viability of PC12 cells co-treated with t-BOOH and nanozymes for two days. **C** Infarct areas of various groups after treatment (n = 4). **D** Neurological scores of different groups with CeO_2_@ZIF-8 therapy for three days (n = 10) [43]. Copyright the Authors 2020

ROS as the signaling messengers play an essential function in the physiological signal transduction pathway [32]. Endogenous and low concentrations of ROS can be produced from normal metabolic processes in the living cells [33, 34]. However, ROS are highly toxic at higher concentrations and can damage protein, RNA, and DNA, leading to cell death [32, 35]. Therefore, ameliorating the oxidative stress induced by ROS has been proved to be beneficial in pathological therapy, such as age-related macular degeneration (AMD), traumatic brain injury, and ischemic disease [23, 36–38]. Nanoceria is often employed as an antioxidant agent to treat ROS-relevant diseases. For instance, AMD is associated with irreversible ROS damage against the macular that may lead to blindness [39, 40]. Generally, evidence showed that AMD pathology can be treated by counteracting the overproduction of ROS. Mitra et al*.* reported a nanoceria with dominated Ce^3+^ that can scavenge the ROS, such as H_2_O_2_ and **·**OH, and inhibit neovascularization formation [41]. Moreover, Yan et al*.* developed a single-atom Pt/CeO_2_ for traumatic brain injury treatment [36]. Compared with CeO_2_, Pt/CeO_2_ exhibits highly enzymatic activity that can scavenge O_2_^•−^, **·**NO, and **·**OH. Furthermore, in vivo administration of Pt/CeO_2_ to C57BL/6 mice can significantly improve the wound recovery by up to 100%, higher than the mice in the untreated group with 50% of the wound closure. Similarly, ischemic stroke is one of the inflammations associated with excessive ROS generation. With excellent antioxidation activity, ceria nanozymes have been applied for efficient treatment of reperfusion-induced injury in ischemic stroke [42]. Although nanoceria with ROS eliminating ability can protect the cells from ROS damage, physiological stability and biocompatibility remained a challenge towards further clinical prospects. Regarding the present issue, He et al. designed a multifunctional nano-system for treating ischemic stroke with prolonged blood circulation time and higher biosafety [43]. CeO_2_@ZIF-8 were prepared by in-situ capping of CeO_2_ with zeolitic imidazolate framework-8 (ZIF-8) (Fig. 2A). In their design, ZIF-8 was served as peroxidase to maintain the activity of CeO_2_, as well as to enhance the penetration and accumulation of CeO_2_ to brain tissue. In PC12 neuronal cells, CeO_2_@ZIF-8 can effectively protect cells from tert-butyl hydroperoxide (t-BOOH) induced cell apoptosis (Fig. 2B). In vivo administration of CeO_2_@ZIF-8 could significantly reduce the infarcted area and increased the neurological scores of mice, confirming that CeO_2_@ZIF-8 can effectively treat mice with ischemic stroke (Fig. 2C, D).

Another interesting application of nanoceria was the elimination of extracellular DNA (eDNA) for anti-biofilms. Biofilms, a community of bacteria cells, prevents a majority of approaches to treat bacterial infectious diseases in humans. eDNA is a crosslinking component of bacteria biofilm, which provides a potential survival benefit for bacterial infection. It has been established that eDNA has a profound impact on the process of biofilm formation. Therefore, eradicating eDNA is an effective strategy to treat biofilm infection. Functionalization of CeO_2_ offers a versatile approach in combating biofilm formation and bacterial infection. Liu et al. designed metal–organic framework (MOF)/Ce-based nanozymes to combat biofilms [44]. The MOF/Ce nanozymes with DNase mimic activities could not only inhibit the biofilm formation but also eradicate established biofilm matrix components by hydrolyzing eDNA. As compared to primitive MOF, MOF/Ce nanozymes possessed higher bactericidal activity via co-incubation. The mechanism of the bactericidal activity of MOF/Ce nanozymes is that two adjacent Ce^4+^ could bind to the oxygen atom of the phosphate group by withdrawing the electrons, resulting in phosphodiester linkage cleaving [45].

## Ferrum-based nanozymes

Ferrum (Fe)-based nanoparticles have gained extensive attention for various biomedical applications due to their magnetic resonance imaging (MRI) performance, POD-mimetic activity, and CAT mimetic properties [46, 47]. Huo et al. reported that Fe_3_O_4_ nanoparticles and GOD co-encapsulated into the mesoporous silica nanoparticles could be effective for tumor catalytic therapy [48]. The intrinsic POD-mimetic activity of Fe_3_O_4_ could generate a considerable amount of **·**OH from H_2_O_2_ produced by the GOD catalysis from glucose. Importantly, the efficiency of **·**OH production efficiency involving the amount of Fe^2+^. Compared with the reaction kinetics of Fe^2+^ with H_2_O_2_ (40–80 L mol^−1^ s^−1^), the reaction rate of Fe^3+^ (9.1 × 10^–7^ L mol^−1^ s^−1^) with H_2_O_2_ is relatively low [49, 50]. In addition, several reports have established links between POD-mimetic activity and CAT mimicking property of Fe_3_O_4_ under various conditions. Gao et al. investigated the catalytic mechanism of Fe^2+^/Fe^3+^ proportion in Fe_3_O_4_ with the use of either a reducing agent (NaBH_4_) or an oxidizing agent (NaIO_4_) [51]. They found that increased Fe^2+^/Fe^3+^ proportion of Fe_3_O_4_ could be achieved by NaBH_4_ treatment, with the correspondingly enhanced peroxidase-like activity of Fe_3_O_4_. On the contrary, decreased proportion of Fe^2+^/Fe^3+^ treated by NaIO_4_ reduces the POD-like activity of Fe_3_O_4_. In addition, the authors showed that the pH, temperature, and dimension of nanoparticles could effectively influence the enzyme-like activity of the Fe_3_O_4_. These pieces of evidence provide insights into the metallic species and their impacts on the enzyme activities of Fe_3_O_4_. Another study by Wang et al. showed that reducing agents in the physiological environment (l-cysteine/NADPH) can restore Fe^3+^ to Fe^2+^ on the surface of Fe_2_O_3_, enhancing the abilities of **·**OH generation by Fe_2_O_3_ [52]. To further understand the impact of the physiological environment, Chen et al. investigated the enzymes-like properties of Fe-oxide (Fe_3_O_4_ and γ-Fe_2_O_3_) nanoparticles at the cellular level by electron spin resonance (ESR) and multi-parameter water quality meter [53]. They demonstrated that Fe_3_O_4_ and γ-Fe_2_O_3_ nanoparticles exhibit higher POD-like activities at pH = 4.8, while CAT-like activities were observed at pH = 7.4. The controllable enzymatic activities in targeted microenvironments provide flexibility and high sensitivity for diverse biomedical applications. Owing to the role of reducing agents in enzyme-like activity, it is feasible that the Fe^3+^ is reduced to Fe^2+^ by the overexpressed GSH in tumor tissues, contributing to the elevation of the ROS generation and resultant tumor destruction.

Fe-based nanozyme with CAT-like activity have been reported to broaden the therapy for ROS-involved cerebral malaria. Zhao et al*.* designed and synthesized recombinant human ferritin (HFn) modified Fe_3_O_4_ (Fenozyme) with blood–brain barrier crossing and ROS-scavenging activity to treat cerebral malaria [54]. From the in vivo murine cerebral malaria experiment, it has been revealed that fenozyme possessed significant ROS scavenging abilities of Fe_3_O_4_ and prominent blood–brain barrier crossing performance of HFn. Administration of the fenozyme can significantly ameliorate the lesion of cerebral malaria and enhance the survival rate of infected mice induced by the parasite.

## Copper-based nanozymes

Copper (Cu) oxide nanomaterials have received significant attention due to their enzyme-mimetic activity [55–57]. The POD-mimetic activity of Cu oxide nanoparticles has focused on ROS production activity by Fenton-like catalysis by Cu^+^ and/or Cu^0^ [58]. Besides, the reaction rate of Cu^+^ with H_2_O_2_ was high than Fe^2+^ because the redox potential of Cu^2+^/Cu^+^ is lower, indicating that Cu^+^ exhibited relatively higher POD-like activities than Fe^2+^ [59, 60]. Similar to Fe^2+^, the Fenton-like activity of Cu-based nanoparticles is the potent antitumor agent. In tumor microenvironment mediated therapy, intratumoral reductive agents (such as GSH) can reduce Cu^2+^ to Cu^+^ species, leading to high selectivity and efficiency. For instance, Ma et al*.* reported copper-amino acid mercaptide nanomaterials (Cu-Cys) with GSH depletion and Cu^+^ production within the tumor microenvironment [60]. After their accumulation at the tumor sites, Cu^+^ species reacted with H_2_O_2_ and produced sufficient ROS, initiating the tumor apoptosis via a Fenton-like reaction. Besides tumor treatment, the POD-like activity of Cu oxide nanoparticles has been employed as antibacterial treatment. Xi et al. designed Cu/carbon nanozymes that can effectively kill Gram-positive and Gram-negative bacteria [61]. Especially, they confirmed that the enzyme-like properties were dependent on the chemical state of Cu. Cu^0^ exhibits high POD-like activities than Cu^2+^ and kill bacteria by Fenton-like reaction under H_2_O_2_-rich environment. The research progress of Cu-based nanozymes shows their promising biomedicine applications in the targeted disease microenvironment.

**Fig. 3:**
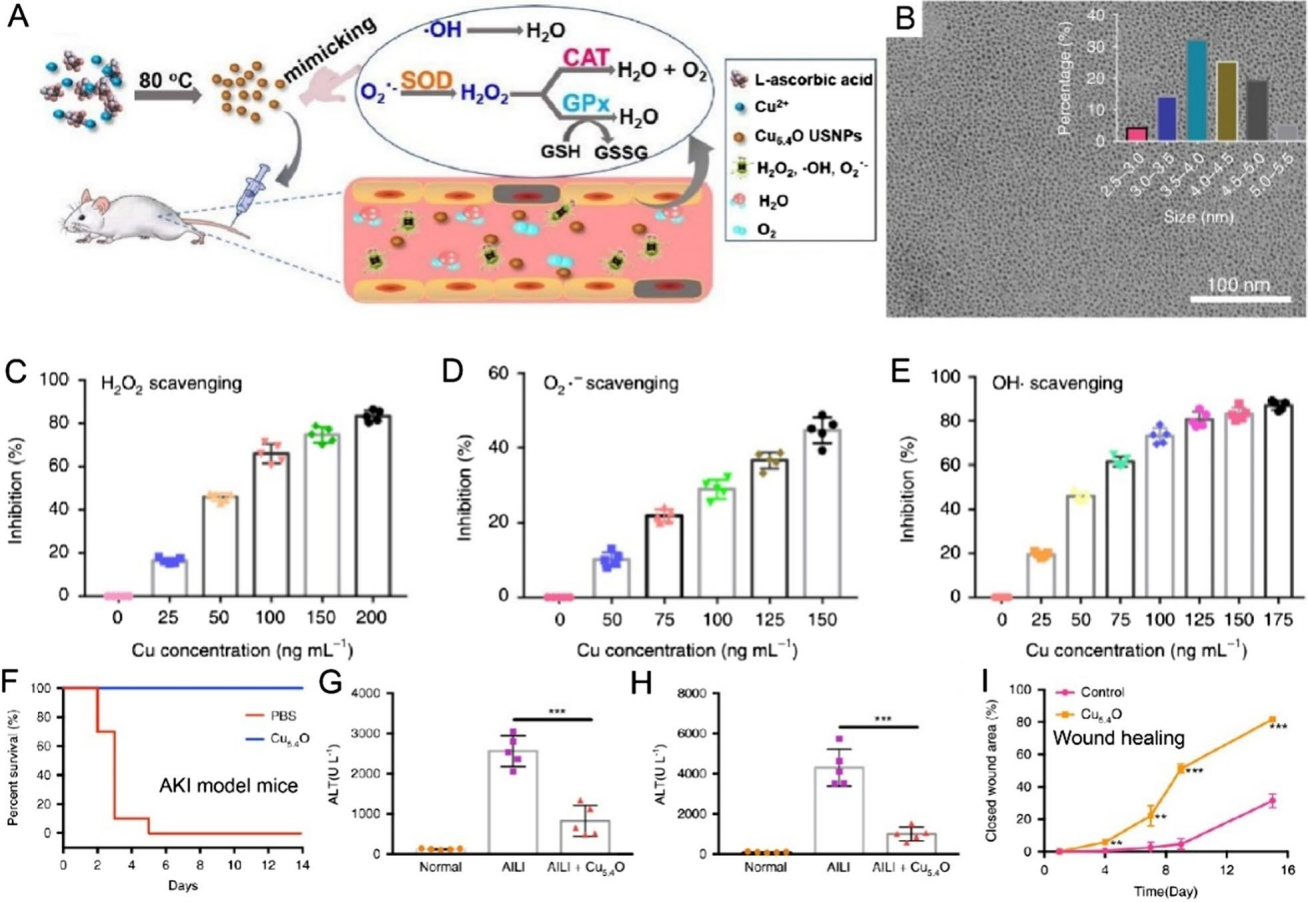
**A** Schematic illustration of the synthesis of ultrasmall Cu_5.4_O nanoparticles with multi enzyme-like properties. **B** TEM image of Cu_5.4_O nanoparticles; **C** H_2_O_2_-, **D** O_2_^•−^-, and **E** **·**OH-scavenging ability of Cu_5.4_O nanoparticles. **F** Survival rate of the AKI model mice after Cu_5.4_O treatment. Detection of **G** serum AST and **H** ALT levels of acetaminophen (APAP)-induced acute liver injury. **I** Wound area of diabetic mice after Cu_5.4_O treatment [67]. Copyright the Authors 2020

Regulating the activity of nanozymes by utilizing an external stimulus may be highly desirable. It has been demonstrated that visible light could modulate the antibacterial activity of CuO. Nurul Karim et al. fabricated a CuO-nanorod that exhibits POD-mimic activities, and the enzymes-like activities were controlled by visible light [62]. Due to the favorable band structure (1.44 eV), CuO exhibit relatively high POD-like efficiency in the presence of light to kill Gram-negative bacteria efficiently. Besides, the Cu_2_O nanoparticles can mimic the cytochrome c oxidase activity [63]. It is worth noting that CuxO nanoparticles synthesized in the presence of the structure-directing agent phenylalanine (Phe) can mimic multienzyme activities, such as GPx, POD, superoxide dismutase (SOD), and catalase, enabling the ROS scavenging performance for Parkinson’s disease amelioration [64]. The potential mechanism is that CuxO could reduce the intracellular ROS levels and alleviating oxidative damages. On the other hand, Korschelt et al*.* reported a copper hydroxide (Cu(OH)_2_) nanoparticle with glycine functionalized (Gly-Cu(OH)_2_) that served as SOD mimics, eliminating O_2_^•−^ radicals generated while smoking [65]. The mechanism of the SOD-like activity by Gly-Cu(OH)_2_ was further investigated. They found that the reduction and re-oxidized by Cu^2+^ of Gly-Cu(OH)_2_ play a prominent role to eliminate O_2_^•−^. Importantly, Lin et al. reported an interesting Cu^2+^-tannic acid (TA) complex nanozyme (Cu-TA) that exhibits SOD-like activity and catalase-like activity for ROS scavenging [66]. The high SOD-like activity of Cu-TA was dependent on the coordination of Cu^2+^ and TA, which enhance the redox potential of Cu^2+^. Additionally, the Cu-TA nanozyme can eliminate **·**OH and decompose H_2_O_2_ to H_2_O. The ROS scavenging efficiency of nanozyme was further investigated after Cu-TA being loaded into the cigarette filter, and the scavenging efficiency was calculated to be 87.0%, 68.9%, and 34.6% of O_2_^•−^, H_2_O_2_, and **·**OH, respectively. To increase therapeutic benefit and reduce systemic toxicity, it is highly desirable to develop the Cu-based nanoparticles with higher antioxidant activity. Other groups also reported multienzymes-like activities of Cu-based nanoparticles, Liu et al. fabricated ultrasmall Cu_5.4_O nanoparticles with extensive ROS scavenging efficiency and abilities to treat ROS-related disease (Fig. 3) [67]. They demonstrated that Cu_5.4_O could exhibit CAT-, SOD-, and GPx-mimicking for enhanced treatment effect against various ROS-mediated diseases at extremely magnitude such as acute kidney injury (AKI) (2 µg/mg for treatment in vivo), liver damage (6 µg /mg for treatment in vivo), as well as wound healing. Moreover, pharmacokinetics and biodistribution experiments revealed that Cu_5.4_O possesses highly renal clearance advances and outstanding biocompatibility.

## Manganese-based nanozymes

Manganese (Mn)-oxide nanoparticles have been demonstrated with intrinsic activities of POD-, GPx-, CAT-, and SOD-like activities due to the variable Mn valent states [68, 69]. In the presence of Cl^–^/HCO^3–^ environment, the Mn^2+^ exhibit POD-like property that can decompose H_2_O_2_ into **·**OH for tumor therapy [9, 70]. Similar to Fe^3+^ and Cu^2+^, the GSH could reduce Mn^3+^/Mn^4+^ into Mn^2+^ in the tumor microenvironment, the depletion of GSH could sensitize the ROS-based therapeutic strategies such as chemodynamic therapy and photodynamic therapy [71–74]. Recently, Fu et al. constructed Mn-doped calcium phosphate nanoparticles with loaded GOD (GOD-MnCaP) for tumor therapy [70]. Under the tumor microenvironment, GOD could catalyze the intracellular glucose into H_2_O_2_ for Mn^2+^-mediated **·**OH generation and gluconic acid for enhanced Mn^2+^-mediated reaction (Mn^2+^  + H_2_O_2_ → Mn^3+^  + **·**OH + OH^−^) [75]. Mn-containing nanomaterials have also been served as CAT-like nanozymes for O_2_ generation, capturing widespread attention in O_2_ mediated therapeutic strategy [72, 76–78]. The Mn-oxide nanoparticles have been established as ROS scavenging agents for the treatment of oxidation-stress mediated diseases [69, 79, 80]. Decreasing ROS levels is one of the important therapeutic mechanisms for Mn-oxidated nanoparticles. However, the targeted delivery strategies remain a great challenge for current Mn-based nanozymes to meet different disease criteria. Based on these challenges, a material design approach may provide an appropriate option to satisfy such demand. Shi et al. reported the Mn_3_O_4_ encapsulated erythrocyte with T7 peptides functionalization system (Mn_3_O_4_@nanoerythrocyte-T7) for ischemic stroke protection [81]. After being accumulated into the infarcted sites via T7 peptides targeting, the Mn_3_O_4_@nanoerythrocyte-T7 can efficiently scavenge the ROS and supply oxygen before thrombolysis stroke, with O_2_ supply to the hemoglobin in the erythrocyte after thrombolysis. Selective targeting to ischemic stroke provides an attractive strategy to achieve a strong Mn-based nanozymes therapeutic effect.

Interestingly, the GOD enzyme-like activity of MnO_2_ nanosheets was reported by Tang et al. [82]. They synthesized MnO_2_ nanosheets (M-NSs) by a one-step wet-chemical method that had high glucose affinity and thermal stability as compared to the natural GOD. Under NIR laser irradiation, M-NSs could achieve the photothermal conversion while the glucose was gradually transformed to gluconic acid and H_2_O_2_, resulting in glucose deprivation enhanced photothermal therapy. Such inorganic nanozyme with GOD-like activity provides a new strategy for the evolution of glucose deprivation and ROS-mediated cancer therapy.

## Molybdenum-based nanozymes

**Fig. 4:**
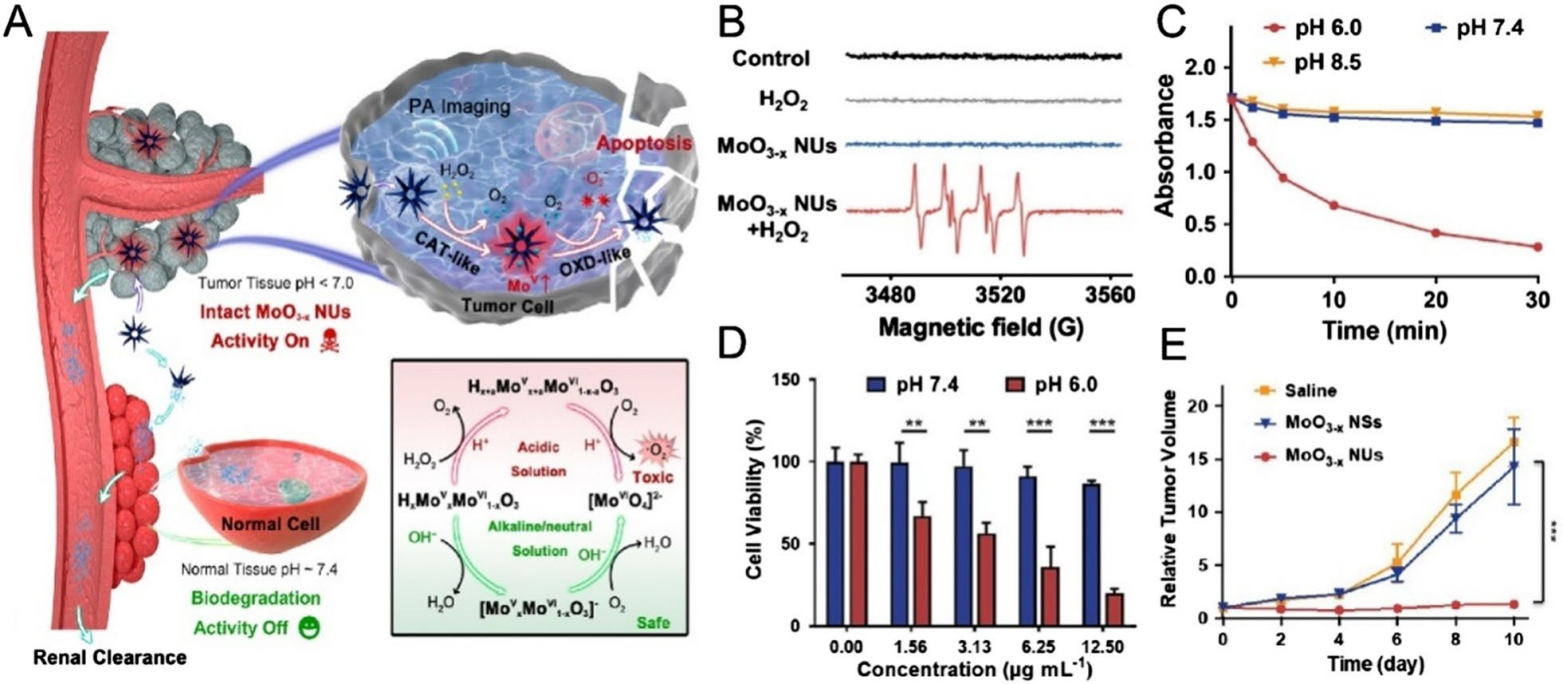
**A** Schematic illustration of the MoO_3-x_ NUs with multi-enzyme mimicking activities for pH-responsive tumor therapy. **B** Electron paramagnetic resonance spectra of MoO_3-x_ NUs in the presence of H_2_O_2_ (1 mM). **C** DPBF degradation curves at different pH conditions (pH 6.0, pH 7.4, and pH 8.5). **D** Cytotoxicity of MoO_3-x_ NUs against B16 melanoma cells at pH 7.4 and pH 6.0; **E** B16 xenograft tumor growth curves after different treatments (n = 5) [89]. Copyright 2019 American Chemical Society

Molybdenum (Mo) nanoparticles have attracted considerable attention as nanozymes [83–85]. There have been many reports regarding the catalytic activity of SOD, CAT, OXD, and sulfite oxidase, etc. [86, 87]. Although Mo-based nanozymes display outstanding enzyme-mimicking performance, it is still difficult to further expand their application to biomedicine. One of the main constraints for the application of Mo-based nanozymes is that these nanozymes carry both antioxidative and oxidative activities simultaneously, and may fail in their application to inhibit oxidative-mediated injury with satisfied outcome. Han et al. synthesized MoO_3-x_ nanodots with CAT- and SOD- mimic activities for Alzheimer's disease treatment [88]. However, the OXD-activities should be considered and may affect the therapeutic benefits of MoO_3-x_. Considering these issues, new strategies should be developed to design intelligent nanozymes with controlled enzyme activity at specific microenvironments with acidity. Hu et al*.* constructed MoO_3-x_ nanourchins (MoO_3-x_ NUs) with pH-dependent multi-enzymatic activity for tumor-specific therapy (Fig. 4) [89]. Under normal physiological pH environment, MoO_3-x_ possessed high biocompatibility due to their stimuli-responsive biodegradation behavior. MoO_3-x_ exhibits excellent catalase enzyme activity under acidic and high H_2_O_2_ conditions such as reduce to the high proportion of Mo^5+^ atoms. Furthermore, MoO_3-x_ exhibits OXD-like activity that could convert O_2_ by disintegrated endogenous H_2_O_2_ to O_2_^•−^ for tumor-specific catalytic therapy. This research provides a new potential therapeutic strategy to reduce the toxicity of nanozymes by controlling their acidic-responsive behavior.

## Cobalt-based nanozymes

**Fig. 5:**
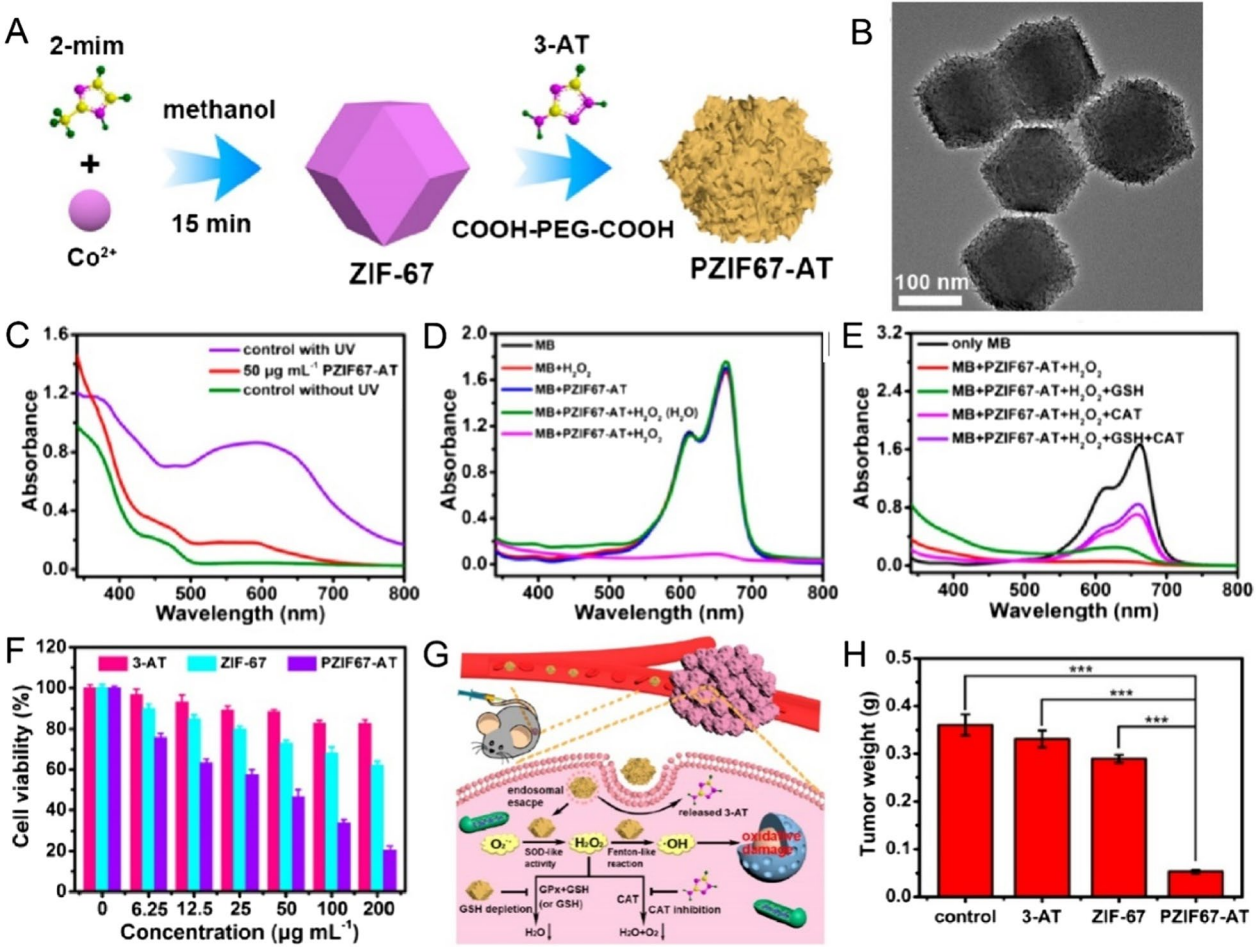
**A** Illustration depicting the preparation of PZIF67-AT nanoparticles. **B** Representative TEM image of PZIF67-AT nanoparticles. **C** UV–Vis absorbance detection of the O^2•−^-eliminated activity of PZIF67-AT. **D** Methylene blue (MB) degradation demonstrated the Fenton-like ability of PZIF67-AT nanoparticles. **E** MB degradation of PZIF67-AT in the presence of CAT or GSH solutions. **F** HeLa cell viability with various treatments. **G** The therapeutic process and mechanism of PZIF67-AT nanoparticles under in vivo conditions [94]. Copyright 2020, American Chemical Society

Intrinsic multienzyme-like activities of cobalt (Co)-based nanoparticles have been reported [90–92]. Dong et al. reported the Co_3_O_4_ possesses the pH-dependent enzyme-like property and the reaction basis is similar to Fe_3_O_4_ [93]. The Co_3_O_4_ showed optimal CAT-like reactivity and SOD-like activity at higher pH conditions (pH ≥ 7.4), and the CAT-like reactivity of Co_3_O_4_ was significantly high than Fe_3_O_4_ at the same conditions. Additionally, Co_3_O_4_ exhibits higher POD-like activity in an acidic medium (pH = 3.6). The catalyzing efficacy of Co-based nanozyme limits their biomedical application. Thus, improving the enzyme activity of nanozymes may be therapeutically attractive for better antitumor efficacy. Recently, Sang et al. developed a polyethylene glycol decorated PZIF67-AT nanoparticles by combining the multienzyme-like activities of Co-based zeolitic imidazole framework-67 and 3-amino-1,2,4-triazole (3-AT) [94]. In their design, the SOD-mimetic activity of PZIF67-AT initially converts O_2_^•−^ to H_2_O_2_ (Fig. 5), subsequently, the production of H_2_O_2_ was converted to **·**OH by PZIF67-AT for cancer therapy. The CAT-like activity of PZIF67-AT was inhibited by 3-AT through binding of the Co-active center. In addition, the overexpressed GSH in TME could also be depleted by PZIF67-AT. This study offers an insight into nanozymes in the applications of tumor therapy.

## Platinum-based nanozymes

Tumor hypoxia and overproduced H_2_O_2_ is the unique feature of solid tumor, which is critical to tumor proliferation and metastasis [95, 96]. Importantly, the therapeutic efficiency of current methods was limited by tumor hypoxia microenvironments, such as photodynamic therapy (PDT) and radiotherapy [97–99]. Unfortunately, oxygen-dependent PDT was severely discouraged due to the low intratumoral oxygen level [100–102]. Fortunately, nanozymes with catalase-like activities could provide a feasible method to improve tumor oxygen-involved therapeutic methods [103, 104]. Based on the catalase-like activity, platinum (Pt) based nanomaterials have been widely applied to decompose the endogenous H_2_O_2_ to O_2_, thus relieving tumor hypoxia for tumor therapy, including PDT and radiotherapy [105, 106]. Zhang et al. report a Pt-PCN-224 nano-platform by decorating Pt on PCN-224 [106, 107]. In the overexpressed H_2_O_2_ microenvironment, Pt is capable of producing O_2_ for the photosensitizer (PCN-224) during the photosensitization process to form ^1^O_2_, which could remarkably enhance the outcome of tumor PDT. Besides, Li et al. synthesized porous Pt nanoparticles that can absorb X-ray and convert H_2_O_2_ to O_2_, improving the radiotherapy efficiency against the malignant tumor [108].

**Fig. 6:**
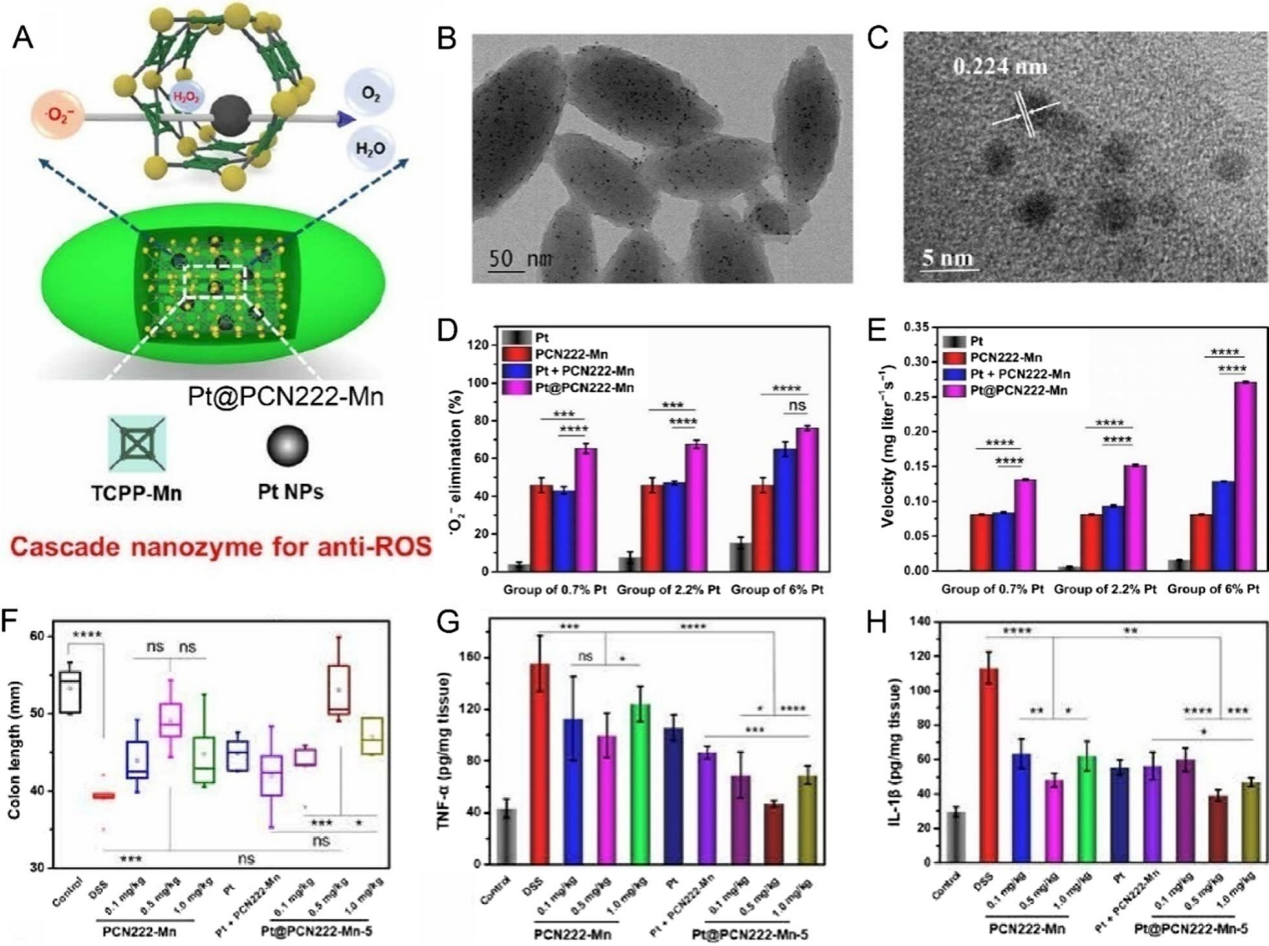
**A** Schematic illustration of the design of Pt@PCN222-Mn. **B** TEM and **C** high-resolution TEM of Pt@PCN222-Mn. **D** O^2–^ elimination ability and **E** oxygen-generated velocities of Pt@PCN222-Mn. **F** the corresponding colon lengths after treatment with various concentrations of Pt@PCN222-Mn. Levels of **G** Inflammatory cytokine IL-1 and **H** TNF-α in colon homogenates after treatment [109]. Copyright the Authors 2020

In addition, Pt-based nanozymes could also be used for ROS and inflammation associated with diseases. In another work, Lin et al. synthesized a cascade nanozyme of Pt@PCN222-Mn to realize anti-inflammatory therapy (Fig. 6) [109]. The PCN222-Mn with SOD-like activity can react with endogenous O_2_^•−^, resulting in superoxide depletion and subsequent H_2_O_2_ generation. Then the Pt nanoparticles exhibited strong CAT-like performance to catalyze H_2_O_2_ for O_2_ generation, thereby benefiting the inflammatory bowel diseases. This research provides the paradigm that the rationally designed nanozymes could have better cascade enzymatic performance against pathologies in a variety.

## Gold-based nanozymes

Enzymatic properties of gold nanoparticles (Au NPs) have widespread uses in biomedical applications [110]. Many studies have reported that Au NPs exhibited multiple-enzymes mimicking abilities such as peroxidase-mimetic activity and glucose oxidase (GOD) activity [111–113]. The GOD-like activity of Au nanoparticles can deplete the glucose and generate H_2_O_2_, which could effectively consume glucose nutrients and inducing cell starving in tumor tissues. For example, Gao et al. synthesized the Au-containing inorganic nanozyme platform (DMSN-Au-Fe_3_O_4_-PEG) [114]. Firstly, Au specifically catalyzes glucose to H_2_O_2_, which was reacted with Fenton agent (Fe_3_O_4_) to produce highly toxic hydroxyl radicals (**·**OH) by typical Fenton reaction for tumor suppression. Additionally, the GOD-mimetic activity of Au nanoparticles has been reported to be synergized with CAT-mimetic nanomaterial enhanced tumor therapy efficiency. Liu et al. loaded Pt and Au into the porphyrin metal–organic frameworks (PCN) with folic acid decoration (P@Pt@P-Au-FA) [115]. The authors showed that endogenous H_2_O_2_ could be catalyzed by Pt to O_2_ for enhanced PDT. The oxygen molecules act as the substrate for gold nanoparticles to convert glucose into H_2_O_2_, supplying the reactant of Pt repeatedly. Within the oxygen cycle, remarkably consumption of glucose and production of gluconic acid could accelerate the catalytic efficiency and the antitumor efficiency of Au. However, the total O_2_ level was not increased during this reaction cycle, low intracellular O_2_ levels still constrain tumor therapies that are oxygen-dependent.

**Fig. 7:**
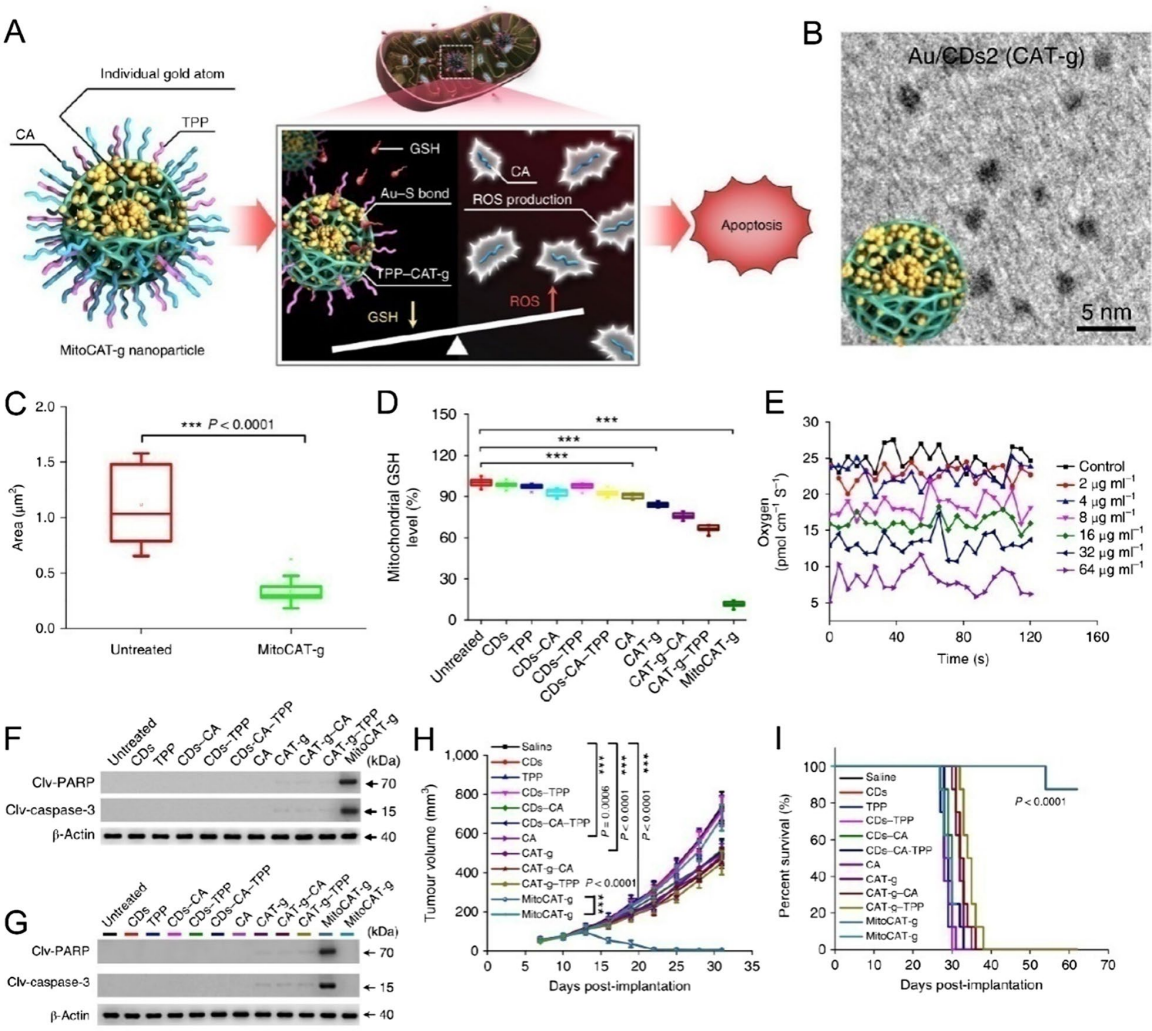
**A** Schematic illustration of the synthesis and therapeutic mechanism of CAT-g. **B** TEM image of MitoCAT-g. Statistics of **C** intracellular mitochondrion area and **D** perimeter of untreated cells and MitoCAT-g-treated cells. **E** The oxygen flux of single HepG-2 cells after treatment with various doses of MitoCAT-g. **G** Western blotting determination of pro-apoptotic proteins (**F**) and (**H**) apoptotic proteins in tumor tissues after different treatments. Relative tumor volumes and **I** survival rate of mice after different treatments [118]. Copyright the Authors 2019

The elevated GSH level in cancer cells enables tumor cells to maintain redox homeostasis and resistance to overexpression of ROS [116]. Depletion of GSH has been developed as a smart strategy for enhanced chemodynamic therapy, chemotherapy, photodynamic therapy, and radiotherapy [117]. However, redox homeostasis destruction has been rarely reported for cancer treatment. Based on the biochemical reactions between Au and thiol of GSH, Gong et al. synthesized single-atom Au nanoagents with GPx-like activity to amplify mitochondrial ROS for tumor therapy (Fig. 7) [118]. First, single-atom Au was incorporated into carbon dot (CAT-g) as metal centers catalysis of GSH. Then, triphenylphosphine and cinnamaldehyde were further employed to modify CAT-g (MitoCAT-g) to enable the mitochondria targeting ability and ROS generating ability. As a result, MitoCAT-g effectively strengthened the oxidative stress in the mitochondrial of tumor cells and trigger apoptosis for cell death.

## Iridium-based nanozymes

Other CAT-like nanozymes containing metal oxide-based nanomaterials, such as iridium (Ir) based nanoparticles, have been developed for biomedical applications [119, 120]. The mechanism of these metal oxide-based nanozymes was associated with the oxidation valent of metal species. For example, Su et al. investigated the connections between the CAT-like property of PVP-Ir(0) NPs and chemical state, demonstrating that the formation of IrO_2_ upon exposure to H_2_O_2_ enables the PVP-IrNPs to exhibit CAT-like activity [121]. The POD activities of PVP-IrNPs were originated from electron transfer mediators. Zhang et al. demonstrated that PVP-IrNPs can scavenge ROS and reactive nitrogen species (RNS) to alleviate AKI [122]. In their work, ultrasmall PVP-IrNPs (1.5 nm) could rapidly accumulate to the kidney after intravenous administration, protecting ROS- or RNS-mediated cellular damage. Furthermore, PVP-IrNPs could be easily excreted to urine by the kidney and exhibit lower systemic toxicity. Besides, Ir-oxide (IrOx) has been reported that acid-activated OXD-like and pH-dependent CAT-like functions for targeted tumor therapies [123]. At neutral normal tissues, the IrOx presented dominantly CAT-like activities. While the POD-like and OXD-like activities were greatly improved along with the gluconic acid generation by GOD catalysis. Importantly, the glutathione (GSH) can be consumed by Ir^4+^, dramatically reduced antioxidative species and enhanced lethality could be ultimately achieved.

## Ruthenium-based nanozymes

**Fig. 8:**
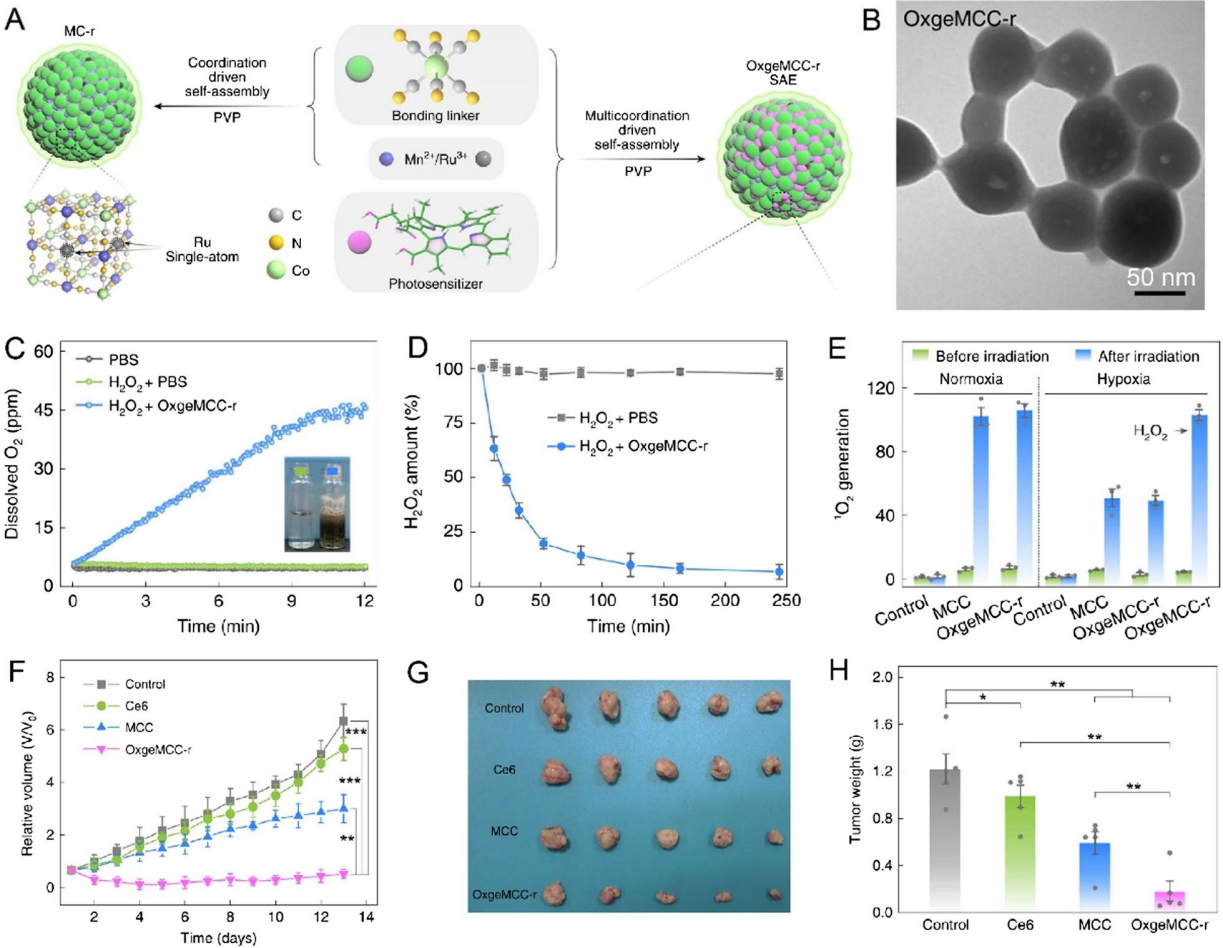
**A** Schematic illustration of the OxgeMCC-r formation process. **B** TEM images of OxgeMCC-r. **C** Time-dependent O_2_ production by OxgeMCC-r with H_2_O_2_. **D** H_2_O_2_ Degradation curve of H_2_O_2_ by OxgeMCC-r. **E** DPBF determines the ^1^O_2_ generation ability of different groups with or without laser irradiation. **F** Tumor volume curves of mice after different treatments. **G** Photo images of tumors collected from different groups after the end of the treatment periods. **H** Tumor weights from different groups of mice after the end of the treatment [126]. Copyright the Authors 2020

Recently, Xu et al. also discovered that ruthenium (Ru)-based nanoparticles with catalase-like activity could be constructed for highly efficient phototherapy against 4-T1 tumors [124]. In their work, RuO_2_@BSA was first prepared by alkaline precipitation methods, and photosensitizer (IR-808-Br_2_) was subsequently decorated into the protein shell to form RuO_2_@BSA@IR-808-Br_2_. First, the RuO_2_ possesses high CAT-like activity, endowing IR-808-Br_2_ with highly efficient PDT activity. Second, the RuO_2_ has photothermal conversion efficiency for PTT. As a result, RuO_2_@BSA@IR-808-Br_2_ achieves sufficient tumor inhibition by synergistically enhanced efficacy of PDT and PTT. The catalase-mimetic activity of RuO_2_ was activated after being exposed to the tumor microenvironment, and immediately convert H_2_O_2_ to oxygenate the IR-808-Br_2_ for the photodynamic process under near-infrared irradiation. Wei et al. reported a multi-functional IrRu-GOD@PEG NPs that could realize tumor starvation therapy and oxidative therapy by chemical catalysis from H_2_O_2_ to ^1^O_2_ [125]. Such an oxidative therapeutic strategy through IrRu alloy nanoparticles provides a new insight for tumor therapy. Recently, the emerging single atom Ru has drawn extensive attention for endogenous O_2_ generation. Wang et al. reported an O_2_ generation single-atom Ru nano-platform (OxgeMCC-r) to enhance the therapeutic efficacy of PDT by self-assembly in the presence of PVP, Mn/Ru, and Ce6 (Fig. 8) [126]. Single-atom Ru allows effective O_2_ generation at a low concentration of the Ru to overcome tumor hypoxia for ^1^O_2_-mediated tumor killing.

## Conclusion and outlook

The fast development and revolution of nanoscience and nanotechnology has broadened extensive research interests for their application in biomedicine. Nanozymes is one of the emerging research frontiers that exhibit great prospects for disease therapy. Herein, we have summarized and discussed the most recent development of nanozymes with their intrinsic therapeutic features for versatile biomedical applications. Despite the tremendous advantages of nanozymes in biomaterial applications, some critical issues and challenges are still needed to be considered. (1) The catalytic efficiency of most nanozymes should be further improved, with controllable enzyme-like activity. It is expected that the enzymatic reactions could be highly lesion site-specific, guaranteeing the biocompatibility and therapeutic specificity. To achieve high enzyme-like activity, introduction of the single-atom nanozymes is the most attractive strategy to achieve such issue, owing to their highly dispersion of the catalytic active sites and atomic utilization efficiency. Yet the loading efficiency of the single metal atoms is limited, challenges for the performance advancement of single-atom nanozymes are remained. In addition, rational design of cascade nanozymes may represent a promising strategy to improve the catalytic efficiency. (2) The molecular mechanism of nanozymes with multienzyme activities are still not clear. For instance, nanozymes mimicking dual-enzymes of CAT and POD, are supposed to exhibit self-competition performance during biomedical applications (as these enzymes both consume H_2_O_2_). The exact molecular mechanism of the electron movements within the metallic species should be further investigated under different conditions. In this regard, disease microenvironment holds great potential to regulate the desired enzymatic performance of specific nanozyme. Numerous studies have demonstrated the feasibility and responsiveness of nanozymes in broad biomedicine applications under characteristic stimuli, such as pH condition, GSH, and light, etc. Another strategy to regulate the specific enzymatic performance of the nanozyme lies in the application of specific inhibitors. (3) The selectivity and specificity of nanozymes should be further improved and optimized. During tumor therapy, although pH- or GSH-responsive nanozymes have exhibited “smart” enzyme-like activities to kill tumor cells, low selectivity and specificity have limited their further applications. To address this dilemma, rationally designed controllable nanozymes are significant to achieve high specificity of its enzyme-mimetic activity against various diseases. Recently, many studies have demonstrated that exogenous stimuli, such as light and ultrasonic, could serve as the trigger to control nanozymes activation. These modalities may provide feasible options to achieve the desired prominent site-specificity. (4) Biocompatibility and biodegradability should be considered. Overcoming the in vivo toxicity of nanozymes during therapeutics is still a barrier toward clinical application. Currently, systemic injection of nanozymes will inevitably cause adverse effect against normal tissues. For metal-based nanozymes, the toxicity is largely associated to the metallic species of the constructed metal-based nanozymes. Although numerous studies have demonstrated the cytoprotective role and biocompatible character of nanozymes, metal ion release is still considered as the possible factor to cause the side impact against normal tissues due to the metal overload. For example, copper or iron overloaded in normal tissue/cells may trigger Fenton or Fenton-like reaction that could severely damage the biomacromolecules as well as the nucleic acids. Therefore, pharmacokinetics of the nanozymes are of critically important during the biocompatibility and biosafety evaluation. The surface tunable properties of nanozymes provide an opportunity to design biosafety agents. Taken into considerations, surface modification is one of the alternative strategies to overcome the limitation of nanozymes. Moreover, considering the ligands of nanozymes could influence therapeutic outcomes, bioavailability, clearance dynamics, and systemic toxicity. From this perspective, it is necessary to carefully choose a suitable ligand and endow nanozymes with higher biosafety.

## Data Availability

Not applicable.

## Abbreviations

GSH: Glutathione
OXD: Oxidase
GOD: Glucose oxidase
POD: Peroxidase
CAT: Catalase
SOD: Superoxide dismutase
GPx: Glutathione peroxidase
TME: Tumor microenvironment
O_2_^•−^: Superoxide anion
H_2_O_2_: Hydrogen peroxide
ROS: Reactive oxygen species
RNS: Reactive nitrogen species
CcO: Cytochrome c oxidase
TNF-α: Tumor necrosis factor alpha
LDH: Lactate dehydrogenase
SPN-C23: Self-regulated nanoceria-doped poly-(cyclopentadithiophene-alt-benzothiadiazole)
PDT: Photodynamic therapy
NIR: Near-infrared
SOSG: Singlet oxygen sensor green
AMD: Age-related macular degeneration
^1^O_2_: Single oxygen species
AMD: Age-related macular degeneration
ZIF-8: Zeolitic imidazolate framework-8
t-BOOH: Tert-butyl hydroperoxide
eDNA: Extracellular DNA
MOF: Metal–organic framework
MRI: Magnetic resonance imaging
ESR: Electron spin resonance
HFn: Human ferritin
Cu-Cys: Copper-amino acid mercaptide nanomaterials
Phe: Phenylalanine
TA: Tannic acid
M-NSs: MnO_2_ nanosheets
MoO_3-x_ Nus: MoO_3-x_ nanourchins
3-AT: 3-Amino-1,2,4-triazole
PCN: Porphyrin metal–organic frameworks
